# Raising the Hat

**Published:** 1882-04

**Authors:** 


### RAISING THE HAT.

IN primitive states the conquered man surrenders himself, his weapons, and whatever of his clothing is worth having ; hence, stripping becomes a mark of submission. Cook, for instance, relates of some Tahitians : "They took off a great part of their clothes and put them on us." In another tribe this ceremony is abridged to the presentation of the girdle only. In Abyssinia, inferiors strip to the girdle before superiors. A further abridgment is found among the natives of the Gold Coast, who salute Europeans by slightly removing their robe from the left shoulder ; but even there special request is shown by completely uncovering the shoulder. In other tribes they also doff the cap. Hence it seems that "the removal of the hat among European peoples, often reduced among ourselves to touching the hat, is a remnant of that process of unclothing himself, by which in early times the captive expressed the yielding up of all he had."

